# Radiological and Not Clinical Variables Guide the Surgical Plan in Patients with Glioblastoma

**DOI:** 10.3390/curroncol31040142

**Published:** 2024-04-01

**Authors:** Carla Martín-Abreu, Helga Fariña-Jerónimo, Julio Plata-Bello

**Affiliations:** 1 Department of Medical Oncology, Hospital Universitario de Canarias, 38320 La Laguna, Spain; 2 Department of Neurosurgery, Hospital Universitario de Canarias, 38320 La Laguna, Spain

**Keywords:** glioblastoma, surgery, extent of resection, biopsy, radiology

## Abstract

*Background and Purpose*: The extent of resection is the most important prognostic factor in patients with glioblastoma. However, the factors influencing the decision to perform a biopsy instead of maximal resection have not been clearly established. The aim of this study was to analyze the factors associated with the intention to achieve maximal resection in glioblastoma patients. *Methods*: A retrospective single-center case-series analysis of patients with a new diagnosis of glioblastoma was performed. Patients were distributed into two groups: the biopsy (B) and complete resection (CR) groups. To identify factors associated with the decision to perform a B or CR, uni- and multivariate binary logistic regression analyses were performed. Cox regression analysis was also performed in the B and CR groups. *Results*: Ninety-nine patients with a new diagnosis of glioblastoma were included. Sixty-eight patients (68.7%) were treated with CR. Ring-enhancement and edema volume on presurgical magnetic resonance imaging were both associated with CR. Corpus callosum involvement and proximity to the internal capsule were identified as factors associated with the decision to perform a biopsy. In the multivariate analysis, edema volume (OR = 1.031; *p* = 0.002) and proximity to the internal capsule (OR = 0.104; *p* = 0.001) maintained significance and were considered independent factors. In the survival analysis, only corpus callosum involvement (HR = 2.055; *p* = 0.035) and MGMT status (HR = 0.484; *p* = 0.027) presented statistical significance in the CR group. *Conclusions*: The volume of edema and proximity to the internal capsule were identified as independent factors associated with the surgical decision. The radiological evaluation and not the clinical situation of the patient influences the decision to perform a biopsy or CR.

## 1. Introduction

Glioblastoma is the most frequent primary malignant neoplasm of the central nervous system (CNS), representing 49.1% of all primary malignant brain tumors and 2% of all cancers [1]. The annual global incidence is estimated to be approximately 308,102 cases (1.6%), with an annual global mortality of 251,329 cases (2.5%) [2]. These tumors are diffusely infiltrative and pleomorphic, with high mitotic activity and microvascular proliferation or necrosis. The prognosis is poor, and survival is usually less than two years [3,4].

Maximal extent of resection (EOR) balanced with functional preservation is the first step in the treatment of glioblastoma. Maximal EOR can be considered as the resection of the entire tumor volume showing contrast enhancement on MRI, including cases where part or all the hyperintense volume on FLAIR surrounding the tumor is resected (supramaximal resection). After surgery, chemoradiation followed by adjuvant chemotherapy has been the standard treatment for two decades [5], with an overall survival (OS) of 27.2% at 2 years and only 9.8% at 5 years [6] with appropriate treatment. Surgical resection remains the cornerstone of glioblastoma treatment. Apart from providing tissue for pathological, genetic, and molecular analyses, maximal EOR has shown the greatest impact on survival in glioblastoma patients. Surgical resection improves the preoperative functional status and decreases the need for high-dose corticosteroids by reducing the mass effect. Maximal EOR has been associated with longer OS, longer progression-free survival (PFS), and improved functional recovery [7,8]. Different intraoperative techniques have been demonstrated to be useful for achieving larger EORs. These techniques include intraoperative imaging [9,10,11,12]; neurophysiological monitoring [13,14]; and fluorescence guidance (5-aminolevulinic acid (5-ALA)) [15]. The use of 5-ALA has increased the number of complete resections (CRs) and has prolonged PFS compared to traditional microsurgical resection [16,17].

There is no clear consensus regarding the EOR threshold associated with a prognostic impact [8,18,19]. One of the first studies on the EOR in glioblastoma was performed using pre- and postsurgical volumetric analyses with MRI by Lacroix et al. [20]. They suggested that resection of at least 98% of the tumor volume was necessary to have an impact on OS. Other studies supporting this hypothesis were performed by Kuhnt et al. [21], Orringer et al. [22], and Grabowski et al. [23], who postulated the need for high EOR percentages to improve OS (>98%, >90%, and >98%, respectively). Chaichana et al. [24] reported a minimum EOR threshold of 70% to have an impact on survival and time to recurrence and introduced a new concept, i.e., residual volume (RV). An RV of 5 cm^3^ was identified as a threshold for impact on survival. Subsequently, Coburger et al. [25] established a lower cutoff at which a significant OS benefit was still obtained, as long as the EOR was at least 60% and the RV was at least 8 cm^3^ [18].

However, in some glioblastoma cases, such as deep tumors, bihemispheric tumors, or patients with a low performance status, only a biopsy is considered. This approach is performed to establish the pathological diagnosis and is not associated with a prognostic benefit for the patient. For multicentric or multifocal glioblastoma, CR is usually not possible; therefore, biopsy is performed instead of surgical excision. Nevertheless, several studies have suggested that surgical cytoreduction may confer increased survival by reducing the mass effect [26,27] and facilitating a local immune response [28,29]. Therefore, the maximum safe EOR that preserves the functional status is recommended when feasible.

Factors influencing the decision to perform a biopsy or to attempt maximal safe resection have been little studied. Tunthanathip et al. [30] reported that a biopsy was usually preferred in cases with corpus callosum involvement. They also suggested that in cases of large glioblastomas in which CR cannot be achieved, maximal resection is not usually attempted [30]. Likewise, significant heterogeneity among surgeons and centers in terms of decision making and selection of treatment modalities in glioblastoma patients has been described [31]. Additionally, clinical guidelines do not facilitate decision making between biopsy or CR and only refer to maximal EOR when feasible. Therefore, the factors that could guide the surgical decision in a glioblastoma patient have not been elucidated. Thus, the aim of this study was to analyze and identify factors associated with the decision to perform a biopsy or surgery with the intention to achieve CR in a glioblastoma patient.

## 2. Methods

### 2.1. Type of Study

This study consisted of a retrospective analysis of a single-center cohort of glioblastoma patients treated by a group of 7 neurosurgeons with a range of 5–20 years of experience.

### 2.2. Subjects

A total of 135 patients with newly diagnosed glioblastoma between January 2015 and June 2021 were identified. The inclusion criteria were (1) male or female subjects at least 18 years of age; (2) pathologically confirmed diagnosis of glioblastoma; (3) availability of presurgical MRI; and (4) initiation of radiochemotherapy after diagnosis. A total of 99 patients fulfilled all these criteria and were finally included in the study. These patients were divided into two groups according to the surgical plan: the biopsy (B) and CR groups. Prior to the decision regarding the type of surgery to be performed, all patients were discussed in a surgical committee comprising the neurosurgeons whose patients were included in the study.

### 2.3. Clinical Variable Evaluation

Clinical and pathological variables were extracted from patients’ medical records. The presurgical functional status was measured with the Karnofsky Performance Status (KPS) scale. This variable was dichotomized in patients with KPS < 70 and patients with KPS ≥ 70. The surgical risk was calculated with the Surgical Risk Calculator of the American College of Surgeons (https://riskcalculator.facs.org/RiskCalculator/, accessed on August 2023).

All patients in the CR group underwent surgery using 5-ALA guidance and intraoperative neurophysiological monitoring. An awake procedure was performed in 6 patients.

### 2.4. Pathological Variable Evaluation

Regarding pathological variables, the MGMT methylation status was evaluated by studying the methylation of the 4 most informative CpG islands in the MGMT promoter. This was conducted in glioblastoma areas dissected from formalin-fixed paraffin-embedded (FFPE) tissue sections using a commercial kit for pyrosequencing (QIAGEN, Hilden, Germany). The cutoff for considering significant methylation was established as 10% of all CpG islands analyzed. For isocitrate dehydrogenase (IDH) mutation evaluation, the most common mutations in IDH1 (R132H) and IDH2 (R172K) were tested by polymerase chain reaction amplification and pyrosequencing. Ki-67 expression analysis was performed by immunochemistry using a validated semiautomatic protocol (uPath, Roche, USA).

### 2.5. Radiological Variable Evaluation

Since magnetic MRI is the preferred diagnostic modality for patients with intracerebral-space-occupying lesions and its findings guide decision making regarding the management of this condition, presurgical MRI features were analyzed in each patient. Two kinds of radiological variables were extracted. Firstly, some imaging features were extracted through a visual inspection by a radiologist and a neurosurgeon. These features included the location of the tumor, subventricular zone involvement, corpus callosum involvement, proximity to the internal capsule, and pattern of contrast enhancement (ring or heterogeneous). Both corpus callosum involvement and proximity to the internal capsule were assessed by jointly analyzing T1-weighted MRI images with and without contrast enhancement, along with T2/T2-FLAIR-weighted MRI images and diffusion tensor imaging (fractional anisotropy map). Secondly, the volumes of contrast enhancement, necrosis, and edema were measured using the online platform OncoHabitats (https://www.oncohabitats.upv.es accessed on 1 August 2023). The necrosis-to-contrast enhancement ratio (NCR) was also calculated for each patient.

All patients underwent postoperative imaging studies: computed tomography in biopsy cases and MRI in cases undergoing attempted complete resection. The EOR was evaluated in the postsurgical MRI (<72 h after surgery) by expert radiologists. CR was considered when more than 99% of the lesion or the total contrast-enhanced area was removed. Tumor progression was considered according to the Response Assessment in Neuro-Oncology (RANO) criteria [32].

### 2.6. Statistics

Statistical analyses were performed using the IBM-SPSS statistical package, version 20.0 for Windows (SPSS, Inc., Chicago, IL, USA). Descriptive statistics (proportions and means ± standard deviations [SDs]) were used to describe the cohort of patients included in the study. Although all continuous variables showed a normal distribution (Kolmogorov-Smirnov, *p* > 0.05), comparisons between the B and CR groups were performed using nonparametric tests (Mann–Whitney U for continuous variables and Fisher’s exact test or chi-square test for discrete variables). Statistical significance was considered when the *p*-value was less than 0.05.

To identify factors associated with the decision to perform a biopsy or CR, uni- and multivariate binary logistic regression analyses were performed. Only those variables that presented a *p*-value < 0.05 in the univariate analysis were included in the multivariate analysis.

Finally, survival analysis was performed in two ways. Firstly, the median OS was estimated for patients in the B group, patients in the CR group, and the whole cohort. OS in the B and CR groups was compared by the log-rank test (*p* < 0.05). Secondly, uni- and multivariate Cox regression analyses of OS were performed in each group of patients. Only those variables that presented a *p*-value < 0.1 in the univariate analysis were included in the multivariate analysis.

## 3. Results

A total of 99 patients (mean age, 61.18 years [SD = 11.45]; 41 women) were included. The CR group comprised 68 patients (68.7%), and the other 31 patients were in the B group. The clinical, radiological, and pathological features are shown in Table 1. Among the patients who underwent surgery with the intention of achieving CR, CR was achieved in 33 patients (48.5%). Resection in the remaining patients in the CR group was considered subtotal.

### 3.1. Comparison between B and CR Patients

Table 2 shows the differences between patients included in the B and CR groups.

The distribution of the tumor location in the cerebral hemispheres was significantly different between groups, more patients with tumors located in the left hemisphere were in the B group (64.5% vs. 54.4%; *p* = 0.045). Furthermore, CR patients presented a higher edema volume on presurgical MRI than B patients (61.3 vs. 42.2 cc; *p* = 0.025) and a higher NCR (0.44 vs. 0.35; *p* = 0.035). In contrast, in a larger percentage of B patients, the tumor showed corpus callosum involvement (54.8% vs. 32.4%, *p* = 0.046) and closer proximity to the internal capsule (67.7% vs. 29.4%, *p* < 0.05). Bearing in mind that the presence of specific neurological symptoms may influence the decision to perform a biopsy or resection in glioblastoma patients, the distribution of the different neurological symptoms between the two groups was specifically studied (Supplementary Table S1); however, no significant differences were identified in this analysis. Finally, as expected, CR patients showed better OS than B patients (15.6 vs. 10.1 months; *p* = 0.002) (Table 2).

### 3.2. Factors Associated with the Selection of Biopsy or Resection

**Figure 1:**
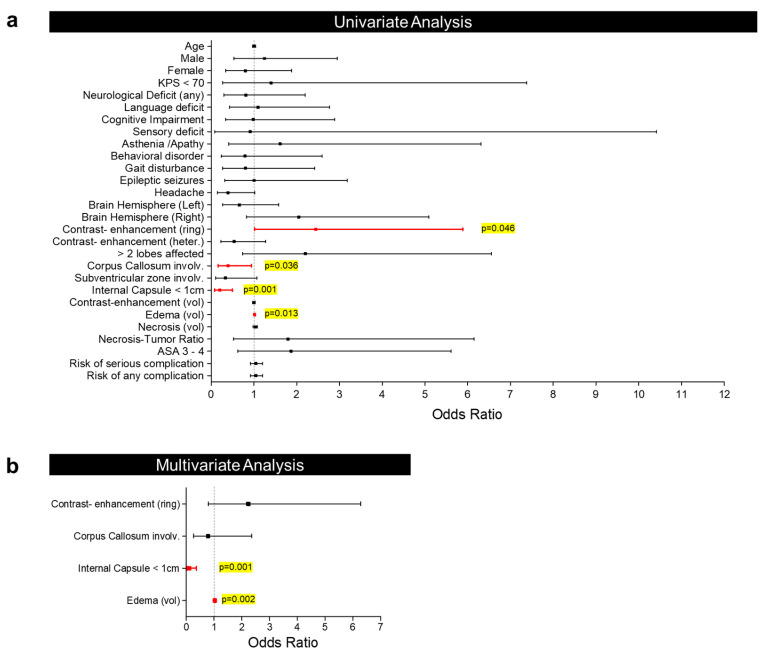
Forest plot showing the binary logistic regression analysis results at the univariate (**a**) and multivariate levels (**b**).

**Figure 2:**
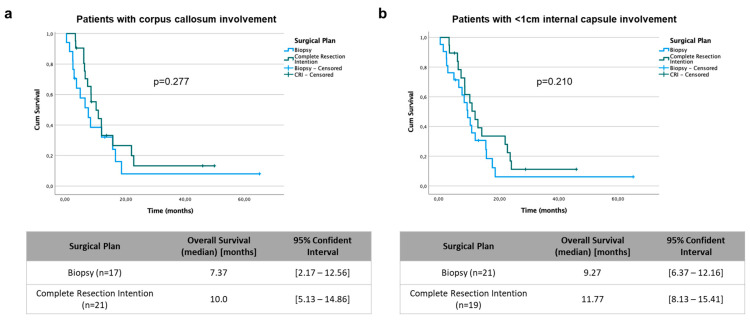
Kaplan–Meier curves for overall survival in patients with corpus callosum involvement (**a**) and in patients with <1 cm internal capsule involvement (**b**). A comparison between biopsy and complete resection intention groups was performed using the log-rank test.

A uni- and multivariate binary logistic regression analysis was performed with the aim of identifying an association between presurgical factors/variables and the decision to perform a biopsy or resection (Table 3). In the univariate analysis, ring enhancement (OR = 2.445; *p* = 0.046) and edema volume (OR = 1.019; *p* = 0.013) were identified as factors associated with the intention to achieve CR. By contrast, corpus callosum involvement (OR = 0.394; *p* = 0.036) and proximity of <1 cm to the internal capsule (OR = 0.198; *p* = 0.001) were identified as factors associated with the decision to perform only a biopsy (Figure 1a). Bearing this in mind, an analysis of only patients with corpus callosum involvement (Figure 2a) and a proximity of <1 cm to the internal capsule (Figure 2b) was performed. The results showed that in such cases, attempting CR did not provide a prognostic benefit. Finally, the multivariate analysis showed that only edema volume (OR = 1.031; *p* = 0.002) and proximity to the internal capsule (OR = 0.104; *p* = 0.001) maintained statistical significance, and therefore, could be considered independent factors associated with the decision to perform a biopsy or resection (Figure 1b).

### 3.3. Factors Associated with Prognosis in the B and CR Groups

Finally, a survival analysis was performed to identify those factors associated with OS. In B patients, the presence of KPS < 70 (HR = 10.319; *p* = 0.011), the volume of contrast enhancement (HR = 1.031; *p* = 0.017), and the volume of edema (HR = 1.012; *p* = 0.081) were considered significant risk factors in the univariate Cox regression analysis (Table 4). Only KPS < 70 (HR = 6.925; *p* = 0.053) was close to reaching statistical significance in the multivariate analysis (Supplementary Table S1).

In contrast, in CR patients, the presence of epileptic seizures (HR = 2.123; *p* = 0.039), tumor location in the right hemisphere (HR = 1.693; *p* = 0.074), corpus callosum involvement (HR = 1.722; *p* = 0.082), and risk of serious complications (HR = 1.072; *p* = 0.096) were associated with worse OS in the univariate Cox regression analysis. The MGMT methylation status (HR = 0.448; *p* = 0.009) showed a significant association with better OS (Table 4). In the multivariate analysis, only corpus callosum involvement (HR = 2.055; *p* = 0.035) and the MGMT methylation status (HR = 0.484; *p* = 0.027) remained statistically significant; thus, they could be considered independent factors associated with prognosis in patients treated with the intention to achieve CR (Supplementary Table S2).

## 4. Discussion

This study analyzed factors associated with the decision to perform a biopsy or surgery with the intention to achieve CR in glioblastoma patients. Radiological instead of clinical features were the most important factors associated with the surgical decision. In this regard, corpus callosum or internal capsule involvement, the edema volume, and contrast enhancement were identified in the univariate analysis. However, only internal capsule involvement and the edema volume maintained statistical significance in the multivariate analysis. Moreover, a survival analysis was performed to identify different risk factors in the B and CR groups. As expected, corpus callosum involvement was associated with a worse prognosis in the CR group. All these findings will be discussed below.

Delving into the differences between patients in the B and CR groups, no statistically significant differences were observed according to age, sex, or clinical presentation. It is important to point out that age was not identified as a determining factor in decision making, which is in line with some publications concluding that the OS in elderly patients is not worse than that in young patients [33,34,35,36]. Interestingly, no association between the surgical intention and the surgical risk was found here, although it is evident that radical surgical resection may not be an option for patients with cardiopulmonary or other comorbidities that limit anesthetic time. Furthermore, no relationship between surgical intention and preoperative KPS or the presence of any neurological symptoms was identified. This is in line with previous reports describing that preoperative KPS impairment is related to symptoms derived from the tumor mass effect; thus, more extensive surgery aids in decompressing the neighboring structures, which may result in KPS improvement [37]. KPS serves as a crucial clinical tool for assessing functional status and guiding treatment decisions in patients with glioblastoma. Throughout the course of treatment, KPS may fluctuate in response to various factors such as tumor progression, treatment-related side effects, and overall disease burden. Understanding how changes in KPS over time influence surgical decision making is paramount. For instance, initial biopsy may be preferred in patients with lower KPS, to minimize surgical risk and maximize quality of life. Conversely, in cases where KPS improves with adjuvant therapies or surgical intervention, the decision for more extensive resection may be warranted to capitalize on potential functional gains and improve long-term outcomes.

Nevertheless, in those cases where there is a high risk of disabling neurological deficits that may limit quality of life, a biopsy is proposed [38]. However, in recent years, the incorporation of new brain mapping techniques has made it possible to aim for CR by minimizing the risk of possible neurological deficits. For this reason, the presence of neurological symptoms is not a factor influencing the decision-making process, which is in line with the observations made in the present work. Neurophysiological monitoring techniques allow brain function to be monitored in real time; thus, proximity to important areas is no longer an obstacle to trying to achieve CR [39]. Thus, the existence of preoperative neurological deficits does not affect the decision to perform CRbut, the location of the tumor and the potential corresponding deficits should be considered when making the surgical decision [40].

Regarding the molecular features, none were associated with the decision to perform a biopsy or intention to achieve CR. This may be expected because in all the included cases the molecular features were known after the surgery; thus, this information could not affect the surgical decision. Furthermore, none of the patients underwent reoperation after the molecular information became available. This approach might be reasonable in cases of an IDH mutation, in which the prognosis is better [41], but only one patient with an IDH mutation was included; thus, no further conclusions can be made. By contrast, the MGMT-promoter-methylation status, a well-known prognostic factor in glioblastoma [42,43,44], was associated with increased OS only in the CR group. In other words, in patients in whom CR has been achieved, MGMT promoter methylation is associated with a better response to alkylating agents [45], but this benefit is affected by the extent of resection.

The distribution of patients according to tumor location was significantly different between the groups. In the B group, most patients had lesions in the left hemisphere, and a large percentage had corpus callosum involvement or very close proximity to the internal capsule. Proximity (<1 cm) and corpus callosum involvement were associated with the decision to perform a biopsy, but only proximity to the internal capsule maintained statistical significance in the multivariate analysis. It is reasonable to think that a location close to the internal capsule would affect the surgeon’s decision, since they would probably not be able to achieve CR without functional consequences, which would lead to the surgeon performing a biopsy. Therefore, corpus callosum involvement or proximity of the tumor to the internal capsule leads to a more conservative approach. Interestingly, analysis of only patients with a proximity of <1 cm to the internal capsule (Figure 2b) and corpus callosum involvement (Figure 2a) indicated that attempting to achieve CR did not provide a prognostic benefit in such cases. The infiltration of the corpus callosum may facilitate increased dissemination of tumor cells, potentially impacting the efficacy of complete surgical resection and, consequently, patient prognosis. In any case, these data should be considered with caution, as they are based on a limited number of patients. In this regard, in cases of butterfly glioblastoma, a rare subtype of bihemispheric tumor which crosses the corpus callosum, surgical treatment remains controversial, and in most cases, only a biopsy is performed [46]. This is probably related to the fact that CR cannot be performed, and the associated morbidity and mortality is very high.

Focusing on other radiological features, ring enhancement and edema volume were identified as factors associated with the intention to achieve CR. Likely, tumors with higher contrast enhancement and larger edema volumes are larger in size, and extensive cytoreductive surgery is the best option to alleviate symptoms related to intracranial hypertension and secondary mass effects. In our study, edema volume maintained statistical significance in both uni- and multivariate binary logistic regression analyses and could be considered an independent factor associated with the decision to perform a biopsy or resection. However, the calculated OR (OR = 1.031) may be insufficient for this factor to be considered clinically significant. The significant difference in the edema volume between the B and CR group patients (Table 2) may explain the results of the regression analysis. In any case, data extracted from the presurgical MRI and not the clinical data were associated with the decision to perform CR or a biopsy. According to these results, a prediction model has recently been reported to support the decision to perform surgical resection or a biopsy in cases of suspected supratentorial glioblastoma, and this prediction model is mainly based on radiological features instead of clinical variables [47].

Some limitations of this study should be considered. The small single-center cohort and the retrospective nature of the study are the main limitations. To mitigate this limitation in future research, prospective cohort studies with standardized data collection protocols, multicenter collaborations, and sensitivity analyses are recommended. Although the number of patients is like other studies, the analysis of a larger and multicenter sample would strengthen the observed results. Further studies are needed to analyze the decision-making process in glioblastoma surgical treatment and to reach a consensus on which surgical treatment it is appropriate to offer to each patient with glioblastoma.

It is noteworthy that, indirectly, the findings of this study demonstrate the significance of multidisciplinary collaboration in glioblastoma management. The participation of neurosurgeons, oncologists, radiologists, and patients in shared decision-making processes or tumor boards is crucial for optimizing treatment strategies and enhancing patient outcomes. By fostering discussions among professionals from diverse specialties and integrating patient preferences and values into treatment decisions, collaborative approaches can result in more informed and individualized care plans. This patient-centered approach not only improves treatment adherence and satisfaction but also contributes to better clinical outcomes.

## 5. Conclusions

In conclusion, our study highlights the critical role of radiological features observed on preoperative MRI in guiding surgical intention for glioblastoma patients. Specifically, proximity to the internal capsule and volume of edema emerged as independent factors influencing the decision between biopsy or resection.

These findings hold significant clinical implications. By prioritizing radiological evaluation over clinical presentation, clinicians can make more informed decisions regarding the optimal surgical approach for individual glioblastoma patients. For instance, patients with tumors in close proximity to the internal capsule may benefit from a more conservative biopsy approach to minimize the risk of neurological deficits, whereas those with extensive edema may warrant aggressive resection to alleviate mass effects and improve outcomes.

Ultimately, our study underscores the importance of integrating radiological assessments into treatment decision-making processes for glioblastoma patients. By leveraging preoperative MRI findings, clinicians can tailor treatment strategies to maximize therapeutic efficacy while minimizing potential risks and complications. Moving forward, further research and clinical validation of these radiological factors are warranted to refine treatment algorithms and optimize outcomes for this challenging patient population.

## Figures and Tables

**Table 1:** Clinical, radiological, and pathological features of the patients included in the study.

Variable		Mean (SD) Count (%)
Age		61.18 (SD = 11.45)
Gender (female:male)		41:58
Karnofsky Performance Status (KPS) ≥ 70		91 (91.9%)
Neurological deficit		74 (74.7%)
Epileptic seizures		16 (16.2%)
Headache		23 (23.2%)
Brain hemisphere	Left	57 (57.6%)
	Right	40 (40.4%
	Bilateral	2 (2.0%)
Contrast-enhancement type	Ring	50 (51.0%)
	Heterogeneous	47 (48.0%)
	No enhancement	1 (1.0%)
>2 lobes affected		27 (27.3%)
Subventricular zone involvement		74 (74.7%)
Corpus callosum involvement		39 (39.4%)
Internal capsule < 1 cm		41 (41.4%)
Contrast-enhancement volume (cc)		21.77 (SD = 15.22)
Edema volumen (cc)		55.32 (SD = 34.78)
Necrosis volumen (cc)		9.39 (SD = 9.92)
Necrosis–tumor enhancement ratio (NTR)		0.41 (SD = 0.41)
ASA	1–2	76 (76.8%)
	3–4	23 (23.2%)
Risk of serious complication (%)		7.22 (SD = 3.44)
Risk of any complication (%)		8.64 (SD = 3.41)
Surgical intention	Biopsy	31 (31.3%)
	Complete	68 (68.7%)
IDH mutation		1 (1.0%)
Ki-67 > 20%		47 (52.2%)
MGMT methylation		57 (58.2%)

**Table 2:** Comparison between patients with different surgical intention (biopsy vs. complete resection).

Variable			Surgical Intention		*p*-Value
			Biopsy (*n* = 31)	Complete Resection (*n* = 68)	
Age			60.9 (SD = 12.98)	61.31 (SD = 10.78)	0.824
Gender (female:male)			14:17	27:41	0.663
Karnofsky Performance Status (KPS)	<70		2 (6.5%)	6 (8.8%)	0.688
	≥70		29 (93.5%)	62 (91.2%)	
Neurological deficit			24 (77.4%)	50 (73.5%)	0.805
Epileptic seizures			5 (16.1%)	11 (16.2%)	1
Headache			11 (35.5%)	12 (17.6%)	0.072
Brain hemisphere	Left		20 (64.5%)	37 (54.4%)	0.045
	Right		9 (29.0%)	31 (45.6%)	
	Bilateral		2 (6.5%)	0	
>2 lobes affected			5 (17.9%	22 (32.4%)	0.213
Subventricular zone involvement			27 (87.1%)	47 (69.1%)	0.080
Corpus callosum involvement			17 (54.8%)	22 (32.4%)	0.046
Internal capsule < 1 cm			21 (67.7%)	20 (29.4%)	0.000
Contrast-enhancement type	Ring		11 (36.7%)	39 (57.4%)	0.070
	Heterogeneous		18 (60.0%)	29 (42.6%)	
	No enhancement		1 (3.3%)	0	
Contrast-enhancement volume (cc)			21.79 (SD = 17.92)	21.76 (SD = 13.96)	0.597
Edema volume (cc)			42.19 (SD = 29.15)	61.30 (SD = 35.67)	0.025
Necrosis volume (cc)			7.45 (SD = 10.42)	10.27 (SD = 9.64)	0.073
Necrosis–tumor enhancement ratio (NTR)			0.35 (SD = 0.52)	0.44 (SD = 0.35)	0.035
ASA		1–2	26 (83.9%)	50 (73.5%)	0.313
		3–4	5 (16.1%)	18 (26.5%)	
Risk of serious complication			6.86 (SD = 2.57)	7.38 (SD = 3.79)	0.765
Risk of any complication			8.29 (SD = 2.55)	8.80 (SD = 3.74)	0.955
IDH mutation			1 (3.2%)	-	0.313
Ki-67 > 20%			19 (67.9%)	28 (45.2%)	0.068
MGMT methylation			16 (53.3%)	41 (60.3%)	0.657
Progression-free survival (months)			6.2 [3.0–9.4]	9.4 [7.9–10.8]	0.068
Overall survival (months)			10.1 [8.3–12.0]	15.6 [13.1–18.2]	0.002

**Table 3:** Uni- and multivariate analysis for determining the factors associated with a surgical intention of total resection.

		Univariate		Multivariate	
Factor		Odds Ratio (95% C.I.)	*p*-Value	Odds Ratio (95% C.I.)	*p*-Value
Age		1.003 (0.967–1.041)	0.870		
Gender	Male	1.251 (0.530–2.950)	0.610		
	Female	0.800 (0.339–1.886)			
Karnofsky Performance Status (KPS) < 70		1.403 (0.267–7.380)	0.689		
Neurological deficit		0.810 (0.298–2.201)	0.680		
Hemiparesis		5.789 (0.713–47.009)	0.100		
Language disorder		1.092 (0.431–2.770)	0.853		
Cognitive impairment		0.985 (0.336–2.890)	0.978		
Sensory deficit		0.909 (0.079–10.419)	0.939		
Asthenia/apathy		1.609 (0.410–6.312)	0.495		
Behavioral disorder		0.793 (0.242–2.598)	0.702		
Gait disturbance		0.804 (0.268–2.416)	0.698		
Epileptic seizures		1.004 (0.316–3.183)	0.995		
Headache		0.390 (0.149–1.022)	0.055		
Brain hemisphere	Left	0.656 (0.273–1.578)	0.347		
	Right	2.048 (0.824–5.091)	0.123		
	Bilateral	-	-		
Contrast-enhancement type	Ring	2.445 (1.015–5.888)	0.046	2.239 (0.798–6.282)	0.126
	Heterogeneous	0.537 (0.227–1.269)	0.157		
	No enhancement	-	-		
>2 lobes affected		2.200 (0.738–6.559)	0.157		
Subventricular zone involvement		0.332 (0.103–1.068)	0.064		
Corpus callosum involvement		0.394 (0.165–0.941)	0.036	0.786 (0.261–2.365)	0.669
Internal capsule < 1 cm		0.198 (0.079–0.496)	0.001	0.104 (0.029–0.372)	0.001
Contrast-enhancement volume (cc)		1.000 (0.972–1.028)	0.993		
Edema volume (cc)		1.019 (1.004–1.034)	0.013	1.031 (1.011–1.051)	0.002
Necrosis volume (cc)		1.033 (0.984–1.084)	0.194		
Necrosis–tumor enhancement ratio (NTR)		1.793 (0.523–6.148)	0.353		
ASA 3–4		1.872 (0.624–5.614)	0.263		
Risk of serious complication		1.048 (0.917–1.198)	0.489		
Risk of any complication		1.048 (0.916–1.198)	0.494		

**Table 4:** Univariate Cox regression analysis for overall survival in both groups of patients.

		Biopsy		Complete Resection	
Factor		Hazard Ratio (95% C.I.)	*p*-Value	Hazard Ratio (95% C.I.)	*p*-Value
Age		1.017 (0.981–1.054)	0.364	1.007 (0.976–1.040)	0.653
Gender	Male	1.397 (0.638–3.061)	0.403	1.145 (0.635–2.067)	0.652
	Female	0.716 (0.327–1.568)		0.873 (0.484–1.575)	
Karnofsky Performance Status (KPS) < 70		10.319 (1.702–62.563)	0.011	0.548 (0.170–1.769)	0.314
Neurological deficit		1.077 (0.428–2.714)	0.875	0.705 (0.388–1.279)	0.250
Epileptic seizures		0.672 (0.200–2.257)	0.520	2.123 (1.040–4.330)	0.039
Headache		0.540 (0.230–1.272)	0.159	1.678 (0.821–3.430)	0.156
Brain hemisphere	Left	1.197 (0.511–2.804)	0.679	0.591 (0.332–1.052)	0.074
	Right	0.716 (0.295–1.737)	0.461	1.693 (0.950–3.016)	0.074
	Bilateral	5.073 (0.557–46.223)	0.150	-	-
Contrast-enhancement type	Ring	1.357 (0.585–3.150)	0.477	0.837 (0.467–1.500)	0.550
	Heterogeneous	0.649 (0.285–1.479)	0.304	1.195 (0.667–2.141)	0.550
	No enhancement	2.069 (0.266–16.071)	0.487	-	-
>2 lobes affected		1.520 (0.545–4.239)	0.424	0.946 (0.509–1.756)	0.860
Subventricular zone involvement		1.512 (0.442–5.170)	0.510	1.300 (0.702–2.407)	0.404
Corpus callosum involvement		1.117 (0.498–2.505)	0.788	1.722 (0.934–3.174)	0.082
Internal capsule < 1 cm		1.065 (0.452–2.505)	0.886	1.596 (0.869–2.933)	0.132
Contrast-enhancement volume (cc)		1.031 (1.006–1.058)	0.017	0.993 (0.972–1.015)	0.522
Edema volume (cc)		1.013 (0.998–1.028)	0.081	1.001 (0.992–1.010)	0.792
Necrosis volume (cc)		1.025 (0.987–1.065)	0.200	0.984 (0.953–1.017)	0.342
Necrosis–tumor enhancement ratio (NTR)		0.496 (0.135–1.822)	0.291	0.570 (0.247–1.312)	0.186
ASA 3–4		1.716 (0.634–4.644)	0.288	1.149 (0.595–2.219)	0.679
Risk of serious complication		1.093 (0.935–1.278)	0.263	1.072 (0.988–1.163)	0.096
Risk of any complication		1.093 (0.930–1.284)	0.282	1.068 (0.983–1.161)	0.121
IDH mutation		0.045 (0.000–324.863)	0.495	-	-
Ki-67 > 20%		1.619 (0.653–4.012)	0.298	1.019 (0.548–1.892)	0.953
MGMT methylation		0.823 (0.374–1.811)	0.628	0.448 (0.246–0.816)	0.009

## Data Availability

Data can be available for other researchers upon reasonable request to the corresponding author.

## Supplementary Materials

The following supporting information can be downloaded at: https://www.mdpi.com/article/10.3390/curroncol31040142/s1, Table S1: Comparison in terms of the type of neurological deficit between patients with different surgical intention (biopsy vs. complete resection); Table S2: Multivariate Cox regression analysis for overall survival in both groups of patients (only variables with a *p*-value < 0.1 in the univariate analysis were included for each group).

## Abbreviations

5-ALA	5-Aminolevulinic acid
B	Biopsy
CNS	Central nervous system
CR	Complete resection
EOR	Extent of resection
HR	Hazard ratio
KPS	Karnofsky Performance Status
MGMT	O(6)-methylguanine-DNA methyltransferase
MRI	Magnetic resonance imaging
NCR	Necrosis-to-contrast enhancement ratio
OR	Odds ratio
OS	Overall survival
PFS	Progression-free survival
RANO	Response Assessment in Neuro-Oncology
RV	Residual volume
SD	Standard deviation
