# Supplementary material for: Radiological and Not Clinical Variables Guide the Surgical Plan in Patients with Glioblastoma
Source: Curr Oncol. 2024 Apr 1;31(4):1899–912. doi: 10.3390/curroncol31040142 (PMC11049408; doi:10.3390/curroncol31040142)
Supplement: Supplementary file 1 [file curroncol-31-00142-s001.zip › curroncol-2894100-supplementary.pdf]

Supplementary material

Supplementary table 1. Comparison in terms of the type of neurological deficit between patients with different surgical intention (biopsy vs. complete resection).

**Variable**	**Surgical intention**		**p-value**
	**Biopsy (n=31)**	**Complete resection (n=68)**	
Hemiparesis	1 (3.2%)	11 (16.2%)	0.097
Language disorder	9 (29.0%)	21 (30.9%)	1
Cognitive impairment	6 (19.4%)	13 (19.1%)	1
Sensory deficit	1 (3.2%)	2 (2.9%)	1
Asthenia /Apathy	3 (14.7%)	10 (14.7%)	0.749
Behavioral disorder	5 (16.1%)	9 (13.2%)	0.759
Gait disturbance	6 (19.4%)	11 (16.2%)	0.776

Supplementary table 2. Multivariate Cox regression analysis for overall survival in both groups of patients (only variables with a p-value < 0.1 in the univariate analysis were included for each group).

		**Biopsy**		**Complete resection**	
**Factor**		**Hazard Ratio (95% C.I.)**	**p-value**	**Hazard Ratio (95% C.I.)**	**p-value**
Karnofsky Performance Status (KPS) < 70		6.925 (0.974 – 49.263)	**0.053**		
Epileptic seizures				2.241 (0.996 – 5.042)	0.051
Brain hemisphere	Left			0.656 (0.358 – 1.203)	0.173
	Right			1.523 (0.831 – 2.793)	0.173
	Bilateral			-	-
Corpus Callosum involvement				2.055 (1.050 – 4.021)	**0.035**
Contrast-enhancement volume (cc)		1.018 (0.982 – 1.055)	0.336		
Edema volumen (cc)		1.007 (0.986 – 1.027)	0.523		
Risk of serious complication				1.060 (0.983 – 1.143)	0.132
MGMT methylation				0.484 (0.254 – 0.923)	**0.027**
